# Recovery of Oil with Unsaturated Fatty Acids and Polyphenols from *Chaenomelessinensis* (Thouin) Koehne: Process Optimization of Pilot-Scale Subcritical Fluid Assisted Extraction

**DOI:** 10.3390/molecules22101788

**Published:** 2017-10-22

**Authors:** Zhenzhou Zhu, Rui Zhang, Shaoying Zhan, Jingren He, Francisco J. Barba, Giancarlo Cravotto, Weizhong Wu, Shuyi Li

**Affiliations:** 1 College of Food Science and Engineering, Wuhan Polytechnic University, Wuhan 430023, China; zhenzhouzhu@126.com (Z.Z.); rui.zhang@utc.fr (R.Z.); zhanshaoying666@126.com (S.Z.); weizhongwuwhpu@126.com (W.W.); lishuyisz@sina.com (S.L.); 2 Nutrition and Food Science Area, Preventive Medicine and Public Health, Food Science, Toxicology and Forensic Medicine Department, Faculty of Pharmacy, Universitat de València, Avda. Vicent Andrés Estellés, s/n, Burjassot 46100, Spain; 3 Dipartimento di Scienza e Tecnologia del Farmaco, University of Turin, Via P. Giuria 9, Turin 10125, Italy; giancarlo.cravotto@unito.it

**Keywords:** *Chaenomelessinensis* (Thouin) Koehne seed oil, subcritical fluid extraction, response surface methodology, fatty acid composition, polyphenol content

## Abstract

The potential effects of three modern extraction technologies (cold-pressing, microwaves and subcritical fluids) on the recovery of oil from *Chaenomelessinensis* (Thouin) Koehne seeds have been evaluated and compared to those of conventional chemical extraction methods (Soxhlet extraction). This oil contains unsaturated fatty acids and polyphenols. Subcritical fluid extraction (SbFE) provided the highest yield—25.79 g oil/100 g dry seeds—of the three methods. Moreover, the fatty acid composition in the oil samples was analysed using gas chromatography–mass spectrometry. This analysis showed that the percentages of monounsaturated (46.61%), and polyunsaturated fatty acids (42.14%), after applying SbFE were higher than those obtained by Soxhlet, cold-pressing or microwave-assisted extraction. In addition, the oil obtained under optimized SbFE conditions (35 min extraction at 35 °C with four extraction cycles), showed significant polyphenol (527.36 mg GAE/kg oil), and flavonoid (15.32 mg RE/kg oil), content, had a good appearance and was of high quality.

## 1. Introduction

Polyunsaturated fatty acids (PUFA), polyphenolics, including bioflavonoids, their derivatives and analogues, are important nutraceuticals and are becoming of increasing nutritional interest [1]. The consumption of dietary PUFA and polyphenols has been shown to reduce the risk of coronary heart disease and cancer, to improve inflammatory conditions, such as arthritis, to reduce plasma triacylglycerol levels and to lower blood pressure [2]. Although PUFA and polyphenols can be found in natural sources, these may not be enough to cover consumer´s requirements. Both food and pharmaceutical products with added PUFA and polyphenols are becoming common in the United States and European Union [3,4,5]. Some previous studies in the available literature have shown that the presence of PUFA and polyphenol-enriched foods on the market, and the promotion of their consumption may significantly increase the level of their intake [6]. These nutraceuticals have been associated with the prevention of some chronic and degenerative diseases [7,8,9]. Therefore, the recovery of these compounds from natural sources for use as food additives and/or nutraceuticals and dietary supplements is an important challenge [10].

*Chaenomelessinensis* (Thouin) Koehne (*C. sinensis*), commonly known as “Guang Pi Mu Gua”, “Chinese-quince” and “Mingzha”, is used in the contemporary food industry for liquors and candies, but has seen thousands of years of use in Chinese medicine [11], for the treatment of rheumatoid arthritis, hepatitis, asthma and the common cold. Most of the benefits of these plants have been attributed to their high content in PUFA, monounsaturated fatty acids (MUFA), and various polyphenolic compounds.

In recent years, the plant has been cultivated in large areas of some regions in China that have converted farmland into forestland and, as a result, yields have rapidly increased, making it an important industrial agricultural crop. Most of the studies on *C. sinensis* have focused on the nutritional characterization and the evaluation of the biological activity of the compounds found within the flesh. However, there is a lack of information on their seeds, which constitute an important part of these plants and have traditionally been seen as waste.

Previous studies have shown that *C. sinensis* seeds are rich in unsaturated fatty acids [12], and polyphenols. Consequently, the reutilization of seeds for functional, edible oil kills two birds with one stone and addresses both the use of waste and by-products and societal health care.

Conventional methods, such as expeller pressing and Soxhlet extraction (SE), have been used to extract plant oils in the food industry. However, these methods require long extraction times at high temperature, while the oil can often contain residual solvent and require heating, thus facilitating rancidity reactions during the following separation process. There has therefore been an increased effort to develop new methods that can improve the oil extraction process and that could potentially allow the extraction yield as well as the nutraceutical properties of food to be modulated.

The last two decades have seen cold-pressed extraction (CPE), microwave-assisted extraction (MAE), and subcritical fluid extraction (SbFE), technologies being proposed as potential tools to improve extraction processes and functional oils of high quality have thus been obtained [2,13,14].

Although a number of subcritical fluids have been used in SbFE, *n*-butane is the most commonly used subcritical fluid mainly because it only requires low critical pressures and temperatures. Moreover, it has an excellent capacity to extract lipophilic compounds [15]. In addition, this solvent is inexpensive, colourless, has a low boiling point and is considered a clean solvent, as no solvent residues remain in the extracted product [8].

The first objective of this work is therefore to optimize SbFE processing conditions (extraction temperature, number of extraction cycles and extraction time), in order to obtain the highest oil yield, as well as the maximal content of unsaturated fatty acids and polyphenols. The response surface methodology will be used for this purpose. Secondly, the potential of SbFE to recover oil from *C. sinensis* seeds will be evaluated and compared with other conventional and innovative extraction methodologies.

## 2. Results and Discussion

### 2.1. Optimization of Processing Conditions for Subcritical Fluid Extraction to Recover Oil from C. sinensis Seeds

Response surface methodology (RSM), with a Box-Behnken design, was used to evaluate and optimize SbFE processing conditions for the recovery of oil from *C. sinensis* seeds. Prior to RSM, a preliminary study was conducted to establish the central point.

**Figure 1:**
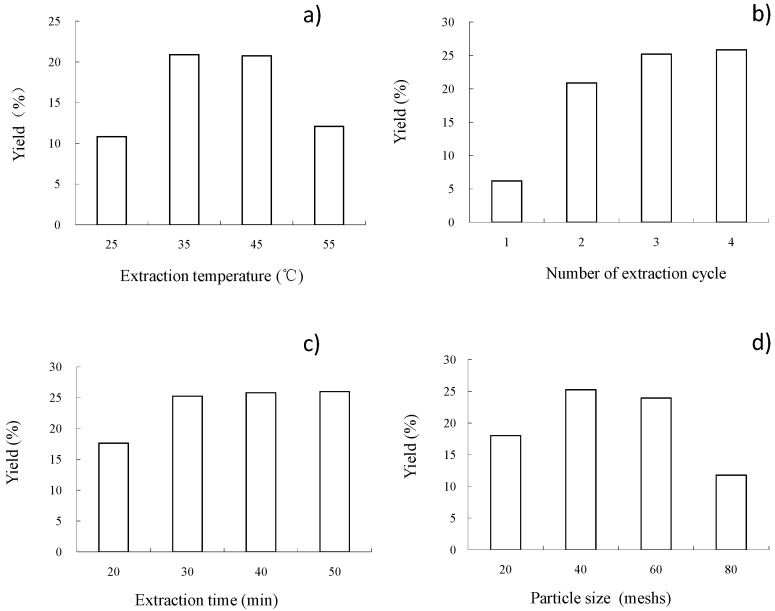
Effects of (**a**) extraction temperature; (**b**) number of extraction cycles; (**c**) extraction time; (**d**) sieve mesh on the yield of *C. sinensis* seed oil extracted using subcritical fluid extraction (SbFE).

A number of temperatures (25, 35, 45 and 55 °C), were evaluated, in order to study the effect of extraction temperature, and the other experimental parameters were set as follows: two extraction cycles, 30 min extraction time, 40 mesh particle size. The results displayed in Figure 1a show that extraction temperatures played an important role in the oil yield from *C. sinensis* seeds. It was observed that the oil yield increased when temperatures were raised to 35 °C, at which temperature the maximum yield was achieved (20.9%). However, a significant decrease in oil yield recovery was found at temperatures of higher than 35 °C. The same trend was observed in the available literature [16], when authors used a solvent/solid ratio of 10 mL/g and took 30 min to extract oil from *C. sinensis* seeds. However, the maximum temperature was 55 °C in that study [16]. Increased temperature promotes a decrease in the density of subcritical *n*-butane, thus hampering oil solubility [17]. Therefore, 35 °C was determined as the centre point for further RSM experiments.

The number of extraction cycles was also evaluated. The number of extraction cycles was set at 1, 2, 3 and 4 for this purpose. The other experimental parameters were set as follows: 35 °C extraction temperature, 30 min extraction time, 40 mesh particle size. As can be seen in Figure 1b, the oil yield recovery from seeds of *C. sinensis* increased when the number of extraction cycles was higher, but there was no significant increase observed after three cycles. Taking into account energy implications, equipment problems and the increase in economic costs, three extraction cycles were therefore selected.

The effect of different extraction times was also investigated. In this case, the extraction time was varied from 20 to 50 min and the other parameters were fixed as follows: 35 °C extraction temperature, three extraction cycles, 40 mesh particle size.

As shown in Figure 1c, oil yield recovery reached the critical value of 25.23% after a span of 30 min and then reached a plateau. Therefore, 30 min was used as the extraction time in the present work as oil yield and the extraction efficiency was taken into consideration. The same extraction time has previously been used [16]. The authors concluded that 30 min was enough time for the extraction of seed oil after SbFE in this study [16].

Finally, the effect of the sieve mesh on SbFE was evaluated. As can be seen in Figure 1d, the oil yield recovery reached a maximum value of 25.23% at a particle size of 40 mesh and a lower oil yield was obtained when the larger sieve mesh was used. This fact can be explained by the inability of the larger sieve mesh samples to form a sheet, thus increasing mass transfer resistance. Consequently, the 40 mesh particle size was selected as the optimal particle size for the next experiments.

### 2.2. RSM Analysis

It was found, based on the analysis of the oil extraction yield and fatty acid profile of *C. sinensis* seed after applying the various methodologies, that SbFE is a promising technology for the recovery of healthy oils that are rich in MUFA and PUFA. SbFE processing conditions were therefore optimized by RSM to improve the oil yield recovery. Box-Behnken experimental design (BBD), with three numeric factors on three levels, was used [18]. Table 1 shows the experimental design scheme and results of the 17 experiments carried out.

The regression models are presented in terms of the actual factors that describe the SbFE process of *C. sinensis* seed oil in Equation (1).

(1) $$\begin{array}{l} Y\;=\;-115.11250\;+\;4.18275X_{1}\;+\;19.86500X_{2}\;+\;1.49850X_{3}\;-\;0.19350X_{1}X_{2}\;-\;\\ 0.010800X_{1}X_{3}\;-\;0.089500X_{2}X_{3}\;-\;0.042650X_{1}^{2}\;-\;1.01000X_{2}^{2}\;-\;0.010850X_{3}^{2} \end{array}$$

The results of significance tests for every regression coefficient and analysis of variance (ANOVA) were analysed using Design Expert software, as shown in Table 2. According to the data in Table 2, the model *F*-value of 76.8 and *p* < 0.0001 implied that the model was highly significant. The “lack of fit *p*-value” of 0.5773 implied that the lack of fit was not significant compared to the pure error, which is desirable. The goodness of fit of the regression model was verified by the determination of correlation coefficient R^2^. The value of R^2^ (0.9900) showed that the model can explain at least 99% of the response value changes. The adjusted R^2^ (0.9771) indicated the significance of the model. It has been suggested that R^2^ should be at least 97.71% for a good fit of the model. Moreover, the low value (3.3%) obtained for the coefficient of variation (CV) showed the precision and reliability of the experimental value [19]. Thus, the quadratic model obtained for the recovery of oil from *C. sinensis* seeds after applying SbFE can be considered adequate to represent the relationship between the response and the independent variables, thus concluding that the experiments were accurate and reliable. Meanwhile, it can be concluded, from the *p*-value of each model term, that the variables with the largest effect were X_2_ (number of extraction cycles), followed by X_1_ (extraction temperature, °C), X_3_ (extraction time, min), and the quadratic term of X_1_×X_1_ (*p*-value < 0.0001). Moreover, the quadratic term X_1_×X_2_ was also found to be significant (*p*-value < 0.01).

**Figure 2:**
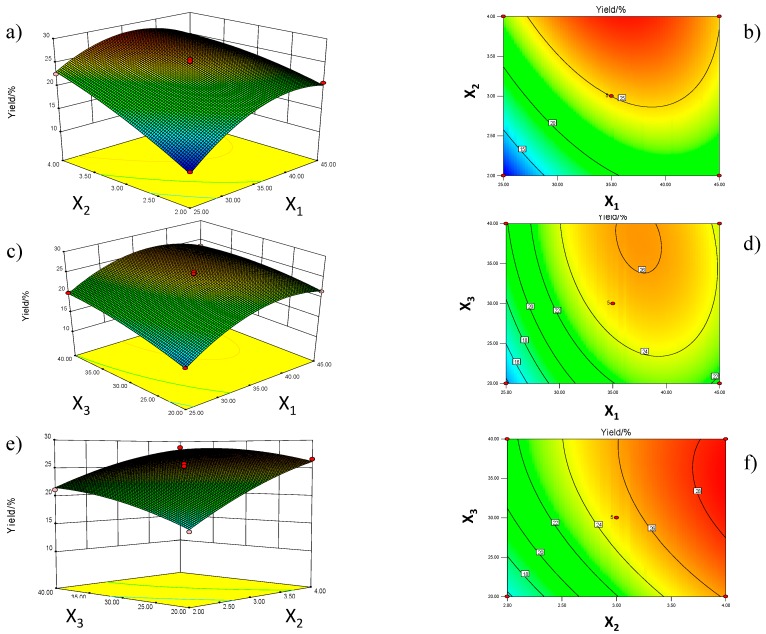
Response surface plots (**a**,**c**,**e**) and contour plots (**b**,**d**,**f**) of oil yield by extraction temperature (*X*_1_), number of extraction cycle (*X*_2_) and extraction time (*X*_3_).

To provide a better visualization of the factors derived from the statistical analysis, three-dimensional (3D), response surface plots and contour plots representing the effects of the independent variables on oil recovery from *C. sinensis* seeds after applying SbFE are shown (Figure 2).

The interactions between two variables and their optimum ranges can be seen [19]. The effects of extraction temperature and number of extraction cycles are displayed in Figure 2a,b. The extraction temperature and number of extraction cycles are directly related to *C. sinensis* seed oil yield. *C. sinensis* seed oil yield increased considerably with the increase in extraction time and decrease in extraction temperature. The interaction between extraction time and particle size is presented in Figure 2c and Figure 2d and extraction temperature had the highest positive effect on the response. However, extraction time produced the lowest impact of the three factors. Figure 2e,f represents the effects of extraction cycle number and extraction time on the yield of *C. sinensis* seed oil. As the number of extraction cycles and extraction time increase, *C. sinensis* seed oil yield also increases. The results above prove that the Box-Behnken design is suitable for the extraction of *C. sinensis* seed oil in this study.

### 2.3. Verification Experiment

The maximum predicted oil yield recovery from *C. sinensis* seeds was 28.66% under the tested conditions (35.54 °C extraction temperature, four extraction cycles and 34.86 min extraction time). The validation experiment was then performed under optimized extraction conditions (35 °C extraction temperature, four extraction cycles and 35 min extraction time), and the *C. sinensis* seed oil yield was actually found to be 29.56 ± 0.28% (*n* = 3). The above results indicate that the Box–Behnken design was able to predict optimum conditions for maximum oil recovery after applying SbFE.

### 2.4. Oil Extraction Recovery from C. sinensis Seeds after Applying Soxhlet, Cold-Pressing, Microwave and Subcritical Fluid Extraction Methodologies

One-way ANOVA was used to evaluate the impact of the Soxhlet (SE), cold-pressing (CPE), microwave (MAE), and subcritical fluid extraction (SbFE) methodologies on the recovery of oil from *C. sinensis*. The ANOVA analysis showed significant differences in the oil recovery of *C. sinensis* seeds after SE, CPE, MAE (ethyl acetate) and SbFE extraction methodologies, observing the highest oil extraction yield (29.0 ± 0.78%) when Soxhlet extraction was used, followed by SbFE (25.79 ± 0.06%), MAE (24.6 ± 0.52%), and CPE (19.0 ± 1.47%). However, as is well-known, SE requires long extraction times and uses large volumes of toxic solvents [20]. As shown in Table 3, significant differences in *C. sinensis* seed oil extraction yield were observed in the four different extraction methods (*p* < 0.05). The data in Table 3 clearly show that the recovery rates of *C. sinensis* seed oil were 88.93%, 84.83% and 65.52% for the three modern extraction techniques (SbFE, MAE and CPE, respectively), taking the SE yield for *C. sinensis* seed oil as the 100% value. Extraction yields demonstrate that SbFE was more effective than MAE (ethyl acetate) and CPE.

### 2.5. Fatty Acid Composition in Oil Recovered from C. sinensis Seeds after Applying Soxhlet, Cold-Pressing, Microwave and Subcritical Fluid Extraction Methodologies

**Figure 3:**
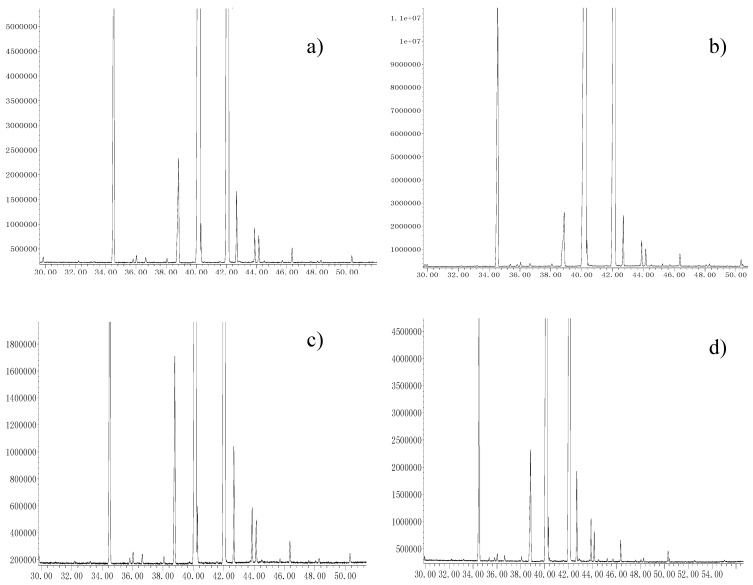
The ion chromatograms of fatty acids of *C. sinensis* seed oil extracted by (**a**) Soxhlet extraction (SE); (**b**) Microwave-assisted extraction (MAE); (**c**) Cold-pressing extraction (CPE); (**d**) Subcritical fluid extraction (SbFE).

As can be seen in Figure 3 and Table 4, ten fatty acids were identified and quantified by using gas chromatography–mass spectrometry (GC–MS) on the various seed oils obtained after applying Soxhlet, cold-pressing, microwave and subcritical fluid extraction techniques. 9-Octadecenoic acid was the predominant fatty acid followed by 9,12-octadecadienoic acid, hexadecanoic acid and octadecanoic acid. Overall, the carbon chain length of the predominant fatty acids in *C. sinensis* seed oil generally ranged from C16 to C22, and unsaturated fatty acids represented over 86% of total fatty acids. This last factor is important to take into account when considering *C. sinensis* seed oil as a functional edible oil with beneficial effects on human health.

One-way ANOVA was conducted in order to evaluate the differences in fatty acid composition in the methodologies used. ANOVA analysis did not show any significant differences in the composition of these fatty acids of SE, CPE and MAE extracts. However, two new fatty acids (dodecanoic acid and tetradecanoic acid) were found, besides the more predominant ones, when SbFE was used. Moreover, significant differences in the relative percentage contents of each component were observed in the four extracts (Table 4). Furthermore, the principal fatty acids found in the seed oil were 9-octadecenoic acid and 9,12-octadecadienoic acid, as also shown in Table 4. The total relative content of these fatty acids in the oil was (85–88%), which was higher than that of peanut oil samples [21]. SbFE led to the highest relative content of monounsaturated fatty acids (46.61%), and unsaturated fatty acids (88.75%).Meanwhile, the content of unsaturated fatty acids in *C. sinensis* oil extracted by SbFE was higher than that of argan oil (81.0–81.7%), and peanut oil [21,22].

In order to quantify the oil extracted from *C. sinensis* seeds after SbFE (which led to the maximum oil yield and a high nutritional and bioactive profile), polyphenol content and physicochemical properties were also evaluated.

The acid value and peroxide value of the SbFE *C. sinensis* seed oil extract were 0.53 mg/g oil and 0.02 mmol/kg oil, respectively. These data demonstrate that the degree of *C. sinensis* seed oil oxidation and free fatty acid content were low in the extraction process. The iodine value was 113.58 g/100 g oil, which suggested that *C. sinensis* seed oil was a semi-drying oil. The high saponification value (185.82 mg/g oil), is similar to the one observed for olive oil (191.93 mg/g oil), suggesting that the main fatty acids found in the *C. sinensis* seed oil present high molecular mass [23,24]. These results are similar to those previously reported by other authors upon evaluating Chinese quince seed oil extracted by SbFE [16]. Moreover, *C. sinensis* seed oil extracted by SbFE was rich in total phenolic compounds (527.36 mg GAE/kg), and total flavonoids (15.32 mg RE/kg), which is an important factor to take into account for the potential use of this oil in food and nutraceutical products.

## 3. Materials and Methods

### 3.1. Materials and Chemicals

*C. sinensis* seeds were provided by Yaorong papaya Bio-Tech Development Co. Ltd. (Shiyan, Hubei, China). Seed samples were dried at 40 °C for 48 h and then stored under darkness at room temperature in polyethylene bags. The dry samples were ground into a powder using a grinder (Tianjin Taisite Instruments Co., Ltd., Tianjin, China), and passed through a 40-mesh sieve just before extraction.

Rutin (>98%), and gallic acid (>98%), were obtained from Chengdu preferred Biological Technology Co., Ltd. (Chengdu, China). Folin–Ciocalteu reagents, purchased from Sigma, were of analytical grade (Sigma-Aldrich Co., Saint Louis, MO, USA). Methanol of chromatographic grade was purchased from Thermo Scientific Co., Ltd. (Waltham, MA, USA). The other chemicals used in the present study were of analytical grade and were purchased from Sinopharm Chemical Reagent Co., Ltd., (Shanghai, China).

### 3.2. Oil extraction Methodologies

#### 3.2.1. Soxhlet Extraction (SE)

Soxhlet extraction (SE) is a traditional method, adopted by the Association of Official Analytical Chemists (AOAC), for extracting fats and oils and thus for lipid determination [8]. The SE of oil from *C. sinensis* seed was performed as described previously with some modifications [13]. Briefly, approximately 4 g of grounded *C. sinensis* seed powder was packed in a Soxhlet apparatus and the oil was extracted using petroleum ether (boiling point: 30–60 °C) at 70 °C for 8 h. When the extraction process was completed, the mixture was evaporated using a BUCHI R-3 rotavapor (BUCHI Labortechnik AG, Flawil, Switzerland) at 38 °C. The oil sample was kept at 4 °C until needed for analysis.

#### 3.2.2. Cold-Pressed Extraction (CPE)

The cold-pressed extraction (CPE) of oil from *C. sinensis* seeds was carried out as previously described by Yang et al. [25]. In brief, 500 g of ground *C. sinensis* seed powder was squeezed three times at room temperature in a CA59G screw type expeller (German Monforts Group, Moenchengladbach, Germany). Subsequently, the pressed oil was centrifuged at 5000 rpm for 10 min in a TGL 205 centrifuge (Changsha Pingfan Instrument Co., Ltd., Changsha, Hunan, China). The extracted oil was kept at 4 °C until needed for analysis.

#### 3.2.3. Microwave-Assisted Extraction (MAE)

The microwave-assisted extraction (MAE) of oil from *C. sinensis* seeds was performed according to the method described by Li et al. [26], with some modifications. Ethyl acetate was selected as the extraction solvent. Generally, ground *C. sinensis* seed powder (4 g) was mixed with 20 mL of extraction solvent in a 100 mL three neck flask. A 500 W microwave apparatus (Uwave-1000, Shanghai SINEO Microwave Chemistry Technology Co., Ltd., Shanghai, China) was used at 30 °C for 30 min at 1200 rpm. After microwave treatment, the mixture was centrifuged at 5000 rpm for 10 min. Subsequently, the supernatant was evaporated using a rotavapor at 38 °C. The oil sample was kept at 4 °C until needed for analysis.

#### 3.2.4. Subcritical Fluid Extraction (SbFE)

**Figure 4:**
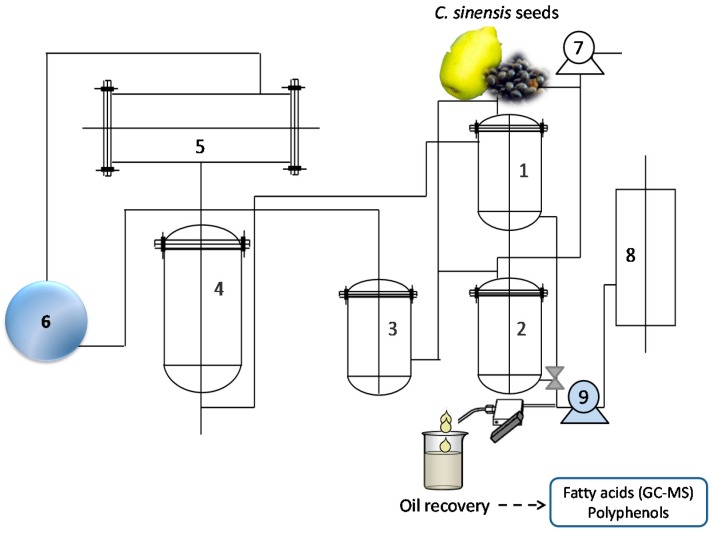
Schematic diagram of the subcritical fluid extraction (SbFE) apparatus and the extraction process followed to recover oil and high-added value compounds from *C. sinensis* seeds. (1) extraction pot; (2) separation pot; (3) surge pot; (4) n-butane storage pot; (5) condenser; (6) compressor; (7) vacuum pump; (8) hot water cylinder; (9) hot water pump.

Subcritical fluid extraction (SbFE), was performed onpilot-scale CBE-10L apparatus (Subcritical Biological Technology Co., Ltd., Shanxi, Henan Province, China). A schematic diagram of the SbFE apparatus is shown in Figure 4. A G445-5/6-13 vacuum pump (Beijing Huizi Mechanical and Electrical Equipment Co., Ltd., Beijing, China) (7) was used to drive the *n*-butane extractant through the system. The maximum extraction capacity was 5 L. Extractor temperature was regulated using a hot water cylinder (8), and hot water pump (9). After extraction, the extractant fluid became gaseous and reached the *n*-butane storage pot (4), via the compressor (6), and condenser (5).

For the SbFE procedure, a 300 g quantity of *C. sinensis* seed powder was mixed in an extraction pot with about 1.5 L of pure *n*-butane (1). In the verification experiment, 1000 grams of *C. sinensis* seed powder were used for each extraction. SbFE was carried out at 35 ± 5 °C, −0.1 MPa for 30 min and then repeated twice. The mixture was immediately pumped into the separation pot (2), after extraction [27]. Oil and solvent were then separated in the separation pot by depressurization from the compressor. The oil was collected and centrifuged at 5000 rpm for 10 min and then stored at 4 °C until needed for analysis.

##### Response Surface Methodology Experimental Design to Optimize the Recovery of Oil Using Subcritical Fluid Extraction

The response surface methodology (RSM) method can be effectively used to display and explain the impact of process variables on extraction yield. It is an effective technique for analysing the interactions between variables and optimizing the processes when multiple variables can influence the output [28,29], thus facilitating the development of new, more efficient and profitable industrial processes. The three variables, extraction temperature (°C, X_1_), number of extraction cycles (X_2_), and extraction time (min, X_3_), were selected at three variation levels to optimize the process using a Box-Behnken design (BBD) (Table 5).

Table 6 details the BBD matrix and response values used to develop the model. The whole design consisted of 17 experimental points carried out in random order. Five replicates (treatments 13–17), at the centre of the design were used to estimate a pure error sum of squares [8].

Regression analysis was performed for the experimental data and was fitted into an empirical second-order polynomial model, as shown in the following Equation (2).

(2) $$Y\;=\;\beta_{0}\;+\;\sum_{i=1}^{3}\beta_{i}X_{i}\;+\;\sum_{i=1}^{3}\beta_{ii}X_{i}^{2}\;+\;\sum_{i=1}^{2}\sum_{j=i+1}^{3}\beta_{ij}X_{i}X_{j}$$

where *Y* is the predicted yield and *X*_1_, *X*_2_, *X*_3_ are three significant independent variables affecting the response. β_0_, β_i_, β_ii_ and β_ij_ are the regression coefficients for intercept, linear, quadratic and interaction terms. The range of independent variables and their levels are shown in Table 5. The response surface methodology data was analysed using Design Expert software (Version 8.0.6.1). The statistical analysis of the model was performed in the form of analysis of variance (ANOVA). Values of *p* < 0.05 were regarded as significant [30].

#### 3.2.5. Calculation of the Oil Extraction Yield and Recovery Rate

The extraction yield (%), was determined gravimetrically as follows [31]:

(3) $$\mathrm{Extraction}\;\mathrm{yield}\;(\%)\;=\;\frac{Mass\;of\;extracted\;oil}{Mass\;of\;dried\;material}\;\times \;100\;$$

Since the SE method was observed to have the highest extraction yield of the four methods, the recovery rate (%) could be calculated as in Equation (4).

(4) $$\mathrm{Recovery}\;\mathrm{rate}\;(\%)\;=\;\frac{Extraction\;yield\;by\;different\;method}{Extraction\;yield\;by\;SE\;method}\;\times \;100$$

### 3.3. Fatty Acid Composition Analysis by GC–MS

Gas chromatography–mass spectrometry (GC–MS) was used to analyse the fatty acid composition of *C. sinensis* seed oil on an Agilent 7890A/5975C GC–MSappliance (Agilent Technologies, Richardson, TX, USA), according to the procedure by Liu et al. [4], with some modifications. Generally, gas chromatographic separation was carried out using an elastic quartz column (SP-2560, 100 m × 250 μm i.d., 0.25 μm film thickness). The GC oven was held at its initial temperature of 100 °C for 1 min, then increased to 230 °C at a rate of 3 °C /min and maintained for 20 min. Helium (purity of 99.99%), was used as the carrier gas at a flow rate of 1.0 mL/min. The injection volume was 1.0 μL at a 10:1 split. The mass spectrometer was operated at 70 eV ionization energy using electron impact ionization (EI), and a scan range from 50 to 550 amu at 2.84 scans/s.

Before GC–MS analysis, the various fatty acids of *C. sinensis* seed oil extracts were derivatized into methyl esters according to Liu et al. [6]. Briefly, 0.03 grams of *C. sinensis* seed oil were added into a 10 mL centrifuge tube and dissolved in 2.5 mL of *n*-hexane and 100 μL of 0.5 mol/L sodium methoxide. The mixture was then blended using an eddy instrument (model IKA-Lab Dancer S25), for 5 min and centrifuged at 4 °C at 5000 rpm for 10 min. Finally, 1.0 μL of the supernatant was injected into the GC–MS system for analysis. The identification of fatty acids was based on matching their recorded retention times and standard mass spectral library (NIST11.L), as provided by the GC–MS software. Compounds with matching rates of over 90% were taken as the target compounds. The normalization method was applied to analyse the fatty acid composition of the oils from the GC peak areas.

### 3.4. Physicochemical Properties and Determination of Polyphenols

Acid value, iodine value, saponification value and peroxide value were determined according to official AOAC methods. Total phenolic content (TPC), was determined according to the Folin–Ciocalteu method [32], with some modifications. Briefly, the reaction mixture, which was composed of 1 mL extract, 0.5 mL of Folin–Ciocalteu reagent, 1.5 mL 20% sodium carbonate and 7 mL distilled water, was stirred and placed in a volumetric flask under darkness for 2 h. The absorbance was then measured at 760 nm. The TPC was calculated according to a standard curve previously prepared with gallic acid as standard. The values were expressed as mg of gallic acid equivalents (GAE), per kg of oil (mg GAE/kg oil). The total flavonoid content (TFC), was determined according to the Al(NO_3_)_3_-NaNO_2_-NaOH colorimetric method [33]. The TFC content was calculated according to a standard curve previously prepared with rutin as the standard. The values were expressed as mg of rutin equivalents (RE), per kg of oil (mg RE/kg oil). The extraction of phenolics and flavonoids from oil for TPC and TFC determination was carried out as follows; one gram of *C. sinensis* seed oil was mixed with 20 mL of *n*-hexane in a 60 mL separating funnel and 45 mL 60% methyl alcohol was then added in three additions with vigorous mixing. The aqueous layer was collected, evaporated and justified to 5 mL with distilled water.

### 3.5. Statistical Analysis

An ANOVA analysis was performed to evaluate the influence of conventional Soxhlet extraction, cold-pressing, microwave-assisted extraction and subcritical fluid extraction on oil recovery and fatty acid profile. Differences at *p* < 0.01 were considered to be significant.

## 4. Conclusions

In comparison with other extraction technologies studied in the present work, SbFE has several advantages. It requires lower operating temperatures and pressures, shorter times and it does not damage heat-sensitive components during processing. SbFE gave higher oil yields with significant polyphenol content. The predominant fatty acids in *C. sinensis* seed oil were 9-octadecenoic acid and 9,12-octadecadienoic acid. In conclusion, SbFE is a safe, efficient extraction technology with which to obtain high-quality, functional oils from *C. sinensis* seeds that are rich in MUFA, PUFA and polyphenols. The developed method is also potentially promising for the high extraction yields of valuable compounds and selective extractions of nutraceuticals that it can provide in the valorisation of plant materials.

## Figures and Tables

**Table 1:** Box-Behnken design for the optimization of SbFE and the values of the observed responses.

Run	Coded Variables			Decoded Variables			Extraction Yield (%)	
	X_1_	X_2_	X_3_	X_1_^a^	X_2_^b^	X_3_^c^	Experimental Value	Predicted Value
1	−1	−1	0	25	2	30	10.84	10.54
2	1	−1	0	45	2	30	20.75	20.26
3	−1	1	0	25	4	30	22.61	23.10
4	1	1	0	45	4	30	24.78	25.09
5	−1	0	−1	25	3	20	13.74	13.65
6	1	0	−1	45	3	20	21.57	21.67
7	−1	0	1	25	3	40	19.93	19.83
8	1	0	1	45	3	40	23.44	23.53
9	0	−1	−1	35	2	20	15.28	15.67
10	0	1	−1	35	4	20	26.56	26.16
11	0	−1	1	35	2	40	21.08	21.48
12	0	1	1	35	4	40	28.78	28.39
13	0	0	0	35	3	30	25.73	25.02
14	0	0	0	35	3	30	23.70	25.02
15	0	0	0	35	3	30	25.23	25.02
16	0	0	0	35	3	30	25.18	25.02
17	0	0	0	35	3	30	25.26	25.02

^a^ *X*_1_ is the extraction temperature (°C); ^b^ *X*_2_ is the number of extraction cycle and ^c^ *X*_3_ is the extraction time (min).

**Table 2:** Analysis of variance (ANOVA) for the regression model.

Parameter	Coefficient Estimate	Standard Error	Sum of Squares	Degree of Freedom	Mean Square	*F*-Value	*p*-Value *
Model			366.00	9	40.67	76.80	<0.0001
Intercept	25.02	0.33		1			
X_1_	2.93	0.33	68.56	1	68.56	129.48	<0.0001
X_2_	4.35	0.26	151.21	1	151.21	285.56	<0.0001
X_3_	2.01	0.26	32.32	1	32.32	61.04	0.0001
X_1_X_2_	−1.94	0.36	14.98	1	14.98	28.28	0.0011
X_1_X_3_	−1.08	0.36	4.67	1	4.67	8.81	0.0209
X_2_X_3_	−0.89	0.36	3.20	1	3.20	6.05	0.0435
X_1_^2^	−4.27	0.35	76.59	1	76.59	144.65	<0.0001
X_2_^2^	−1.01	0.35	4.30	1	4.30	8.11	0.0248
X_3_^2^	−1.08	0.35	4.96	1	4.96	9.36	0.0183
Residual			3.71	7	0.53		
Lack of fit			1.33	3	0.44	0.75	0.5773
Pure error			2.37	4	0.59		
Cor Total			369.71	16			
R^2^ = 0.9900				Adj R^2^ = 0.9771			
C.V.% = 3.30				Pred R^2^ = 0.9323			
PRESS = 25.03				Adeq precision = 31.988			

* *p*-Value < 0.01 highly significant; 0.01 < *p*-value < 0.05 significant; *p*-value > 0.05 not significant. *X*_1_ is the extraction temperature (°C), *X*_2_ is the number of extractions, and *X*_3_ is the extraction time (min).

**Table 3:** The yield and recovery rate of *C. sinensis* seed oil extracted using the different methods.

Method	Extraction Yield (%)	Recovery Rate (%)
SE	29.0 ± 0.78 ^a^	100.00
SbFE	25.79 ± 0.06 ^b^	88.93
MAE (ethyl acetate)	24.6 ± 0.52 ^c^	84.83
CPE	19.0 ± 1.47 ^d^	65.52

Different letters in the same column represent significantly different mean values (*p* < 0.05). SE: Soxhlet extraction. SbFE: Subcritical Fluid Extraction. MAE: Microwave-assisted extraction. CPE: Cold-pressing extraction. (mean ± SD, *n* = 3).

**Table 4:** Fatty acid composition and their relative percentages in *C. sinensis* seed oil as extracted by the four different methods.

Fatty Acids, Methyl Ester	Abbreviated Formula	Characteristic Ions	Molecular Weight	Relative Percentage (%)			
				SE	MAE	CPE	SbFE
Dodecanoic acid	C_12:0_	74, 171, 214	200	-	-	-	0.01
Tetradecanoic acid	C_14:0_	74, 199, 242	228	-	-	-	0.02
Hexadecanoic acid	C_16:0_	74, 227, 270	256	9.76	8.36	9.61	11.21
Octadecanoic acid	C_18:0_	74, 255, 298	284	2.22	2.24	2.22	-
9-Octadecenoic acid	C_18:1_	55, 264, 296	282	43.81	44.65	44.38	46.09
9,12-Octadecadienoic acid	C_18:2_	67, 263, 294	280	42.45	42.7	42.53	41.79
9,12,15-Octadecatrienoic acid	C_18:3_	69, 261, 292	278	0.33	0.33	-	0.35
Eicosanoic acid	C_20:0_	74, 283, 326	312	0.85	1.02	0.85	-
11-Eicosenoic acid	C_20:1_	55, 292, 324	310	0.39	0.46	0.41	0.52
Docosanoic acid	C_22:0_	74, 143, 354	340	0.19	0.23	-	-
Monounsaturated fatty acids (MUFA)				44.20	45.11	44.79	46.61
Polyunsaturated fatty acids (PUFA)				42.78	43.03	42.53	42.14
Unsaturated fatty acids				86.99	88.14	87.32	88.75

- not detected. Soxhlet extraction (SE), Microwave-assisted extraction (MAE), Cold-pressing extraction (CPE), Subcritical fluid extraction (SbFE).

**Table 5:** Variables and experimental design levels for response surface analysis.

Symbol	Independent Variable	Coded Levels		
		−1	0	1
X_1_	Extraction temperature (°C)	25	35	45
X_2_	Number of extraction cycle	2	3	4
X_3_	Extraction time (min)	20	30	40

**Table 6:** Box-Behnken design for the optimization of SbFE and the values of observed responses.

Run	Coded Variables			Decoded Variables			Extraction Yield (%)	
	X_1_	X_2_	X_3_	X_1_^a^	X_2_^b^	X_3_^c^	Experimental Value	Predicted Value
1	−1	−1	0	25	2	30	10.84	10.54
2	1	−1	0	45	2	30	20.75	20.26
3	−1	1	0	25	4	30	22.61	23.10
4	1	1	0	45	4	30	24.78	25.09
5	−1	0	−1	25	3	20	13.74	13.65
6	1	0	−1	45	3	20	21.57	21.67
7	−1	0	1	25	3	40	19.93	19.83
8	1	0	1	45	3	40	23.44	23.53
9	0	−1	−1	35	2	20	15.28	15.67
10	0	1	−1	35	4	20	26.56	26.16
11	0	−1	1	35	2	40	21.08	21.48
12	0	1	1	35	4	40	28.78	28.39
13	0	0	0	35	3	30	25.73	25.02
14	0	0	0	35	3	30	23.70	25.02
15	0	0	0	35	3	30	25.23	25.02
16	0	0	0	35	3	30	25.18	25.02
17	0	0	0	35	3	30	25.26	25.02

^a^ *X*_1_ is the extraction temperature (°C); ^b^ *X*_2_ is the number of extractions; and ^c^ *X*_3_ is the extraction time (min).
